# Efficacy and Safety of Lithium Treatment in SARS-CoV-2 Infected Patients

**DOI:** 10.3389/fphar.2022.850583

**Published:** 2022-04-14

**Authors:** Carlos Spuch, Marta López-García, Tania Rivera-Baltanás, J. J Cabrera-Alvargonzález, Sudhir Gadh, Daniela Rodrigues-Amorim, Tania Álvarez-Estévez, Almudena Mora, Marta Iglesias-Martínez-Almeida, Luis Freiría-Martínez, Maite Pérez-Rodríguez, Alexandre Pérez-González, Ana López-Domínguez, María Rebeca Longueira-Suarez, Adrián Sousa-Domínguez, Alejandro Araújo-Ameijeiras, David Mosquera-Rodríguez, Manuel Crespo, Dolores Vila-Fernández, Benito Regueiro, Jose Manuel Olivares

**Affiliations:** ^1^ Translational Neuroscience Research Group, Galicia Sur Health Research Institute (IIS-Galicia Sur), SERGAS-UVIGO, CIBERSAM, Vigo, Spain; ^2^ Department of Psychiatry, Hospital Álvaro Cunqueiro, SERGAS, Vigo, Spain; ^3^ Microbiology and Infectology Research Group, Galicia Sur Health Research Institute (IIS Galicia Sur), SERGAS-UVIGO, Vigo, Spain; ^4^ US Navy Medical Corps Commander, Medical Director at Educational Alliance, Medical Director at Rejuvenation Health, New York, NY, United States; ^5^ Picower Institute for Learning and Memory, Massachusetts Institute of Technology, Norfolk, United States; ^6^ Universidade de Vigo, Vigo, Spain; ^7^ Infectious Diseases Unit and Virology & Pathogenesis Group, Department of Internal Medicine, Hospital Álvaro Cunqueiro, Galicia Sur Health Research Institute (IIS-Galicia Sur), SERGAS-UVIGO, Vigo, Spain; ^8^ Intensive Care Unit, Critical Care and Emergency Department, Hospital Álvaro Cunqueiro, SERGAS, Vigo, Spain; ^9^ Microbiology Department, Hospital Álvaro Cunqueiro, SERGAS, Vigo, Spain; ^10^ Microbiology and Parasitology Department Medicine and Odontology, Universidade de Santiago, Santiago de Compostela, Spain

**Keywords:** COVID-19, SARS-CoV-2, clinical trial, inflammation, lithium carbonate

## Abstract

At the beginning of the pandemic, we observed that lithium carbonate had a positive effect on the recovery of severely ill patients with COVID-19. Lithium is able to inhibit the replication of several types of viruses, some of which are similar to the SARS-CoV-2 virus, increase the immune response and reduce inflammation by preventing or reducing the cytokine storm. Previously, we published an article with data from six patients with severe COVID-19 infection, where we proposed that lithium carbonate could be used as a potential treatment for COVID-19. Now, we set out to conduct a randomized clinical trial number EudraCT 2020–002008–37 to evaluate the efficacy and safety of lithium treatment in patients infected with severe SARS-CoV-2. We showed that lithium was able to reduce the number of days of hospital and intensive care unit admission as well as the risk of death, reduces inflammatory cytokine levels by preventing cytokine storms, and also reduced the long COVID syndromes. We propose that lithium carbonate can be used to reduce the severity of COVID-19.

## Introduction

Since COVID-19 (coronavirus infectious respiratory disease 2019) and the responsible virus, SARS-CoV-2 (severe acute respiratory syndrome coronavirus 2), were described in December 2019, there has been no effective antiviral treatment for patients. Worldwide, this disease has affected more than 213 million people and has already killed more than 4 million (WHO, August 2021 data).

Clinical manifestations of COVID-19 range from asymptomatic infection to bilateral pneumonia, acute respiratory distress syndrome (ARDS), cytokine storm, coagulopathy, thrombosis, and other extrapulmonary syndromes (Woolhouse et al., 2012). Although this infection is characterized by respiratory symptoms, numerous studies have shown that patients infected with SARS-CoV-2 can have other organs such as the heart, kidneys, gastrointestinal tract, brain, and liver affected (Oldstone et al., 1993).

This pandemic is currently being combated by immunizing the population with different vaccines developed in many countries. However, focusing the strategy only on vaccination is showing many weaknesses: the constant appearance of new variants, the large number of people who refuse vaccines worldwide, the difficult access to the vaccine for a large part of the world population, and the percentage of people who once infected suffer a rapid deterioration of their health and end up in hospital. This scenario is indicating to us the importance of developing effective treatments for those infected with COVID-19 that will help us in the management of this disease.

Therefore, a potent antiviral agent that can be administered in the early stages of infection is urgently needed. In the absence of a specific treatment against this new virus, different drugs have been tested since the beginning of the pandemic, such as hydroxychloroquine and chloroquine, two antimalarial agents (Antia et al., 2003; Greenwood, 2014). Treatment with hydroxychloroquine was shown to be of no benefit and was associated with increased mortality in patients with COVID-19 (De Clercq and Li, 2016). Another treatment used in many patients was remdesivir (GS-5734) (Wang et al., 2020), a viral RNA polymerase inhibitor that had demonstrated *in vitro* antiviral activity against SARS-CoV-1 and MERS-CoV (Cunningham et al., 2020). However, this treatment showed no clinical benefit in hospitalized patients (Cascella et al., 2022; Gao et al., 2020). The same was true for the lopinavir/ritonavir combination, a treatment used for HIV prevention. Although lopinavir had demonstrated *in vitro* activity against SARS-CoV-2 (Hao et al., 2015; Duvall and Gallicchio, 2017), ex-post clinical trials showed that the lopinavir/ritonavir combination also had no clinical benefit in hospitalized COVID-19 patients (Hao et al., 2015; Zhao et al., 2020).

Other immune-modulating treatments have been administered in some patients, such as TNFα inhibitors that block the interaction of the cytokine with its receptors p55 or p75 (infliximab, adalimumab, certolizumab) (Ayuso, 1994; Chen et al., 2015; Cui et al., 2015; HJ et al., 2018). Some monoclonal antibodies, such as anti-IL6 (tocilizumab) or anti-Receptor IL6 (sarilumab) showed in a recent meta-analysis a slight decrease in 28-days mortality (Karampitsakos et al., 2021; Mariette et al., 2022), and more recently, sotrovimab, a SARS-CoV-2 neutralizing antibody, showed a reduction in the risk of disease progression (Gupta et al., 2021). Thus, with these types of biologic therapies, it has been concluded that these drugs should be used in specific types of patients, such as individuals with very high levels of IL-6. Finally, the recovery study concluded that, of all the therapies tested, dexamethasone 6 mg for 7–10 days was the one that showed the best results and is the currently accepted therapy in all hospitals (Horby et al., 2021; Munch et al., 2021).

The COVID-19 pandemic has highlighted the lack of effective antiviral compounds. Paxlovid (Pfizer), the first antiviral drug for COVID-19, has recently been approved. Paxlovid reduces the chance of hospitalization or death by 89% (Mahase, 2021). Molnupiravir (developed by Merck) has been shown to be highly effective in reducing infectious virus and SARS-CoV-2 viral RNA in the nasopharynx, reducing the risk of hospitalization and death by approximately 50% compared with placebo for patients with mild or moderate COVID-19 (Holman et al., 2021; Willyard, 2021). These new drugs open up the possibility for patients to be treated at home, with a combination of a capsule and a pill, but cost and access to these drugs remain serious obstacles. The continuing rise in hospitalizations and deaths following the approval of these drugs demonstrates this.

Since the beginning of the pandemic, we observed that lithium carbonate had a positive effect on the recovery of severely ill patients with COVID-19. In 2020, we published a case report article with data from six patients with severe COVID-19 infection, where we proposed that lithium carbonate could be used as a potential treatment for COVID-19 (Shorter, 2009; Rosen, 2017; Spuch et al., 2020). In that study, we showed how treatment with lithium carbonate reduced inflammation levels and rapidly increased lymphocyte levels, ostensibly improving the health of these patients compared to patients under the standard treatment used at that time.

Lithium was approved by the FDA in 1970 as a psychotropic antidepressant/mood-stabilizing drug to treat bipolar affective disorders (Won and Kim, 2017). However, lithium is also known to be involved in cellular processes such as apoptosis, proliferation, motility, gene expression, glycogen synthesis and inflammation. Recently, evidence has accumulated that lithium can also inhibit infection by DNA- and RNA-type viruses, such as HIV (human immunodeficiency virus), HSV (herpes simplex virus), infectious bronchitis virus, canine parvovirus, porcine parvovirus, pseudorabies herpesvirus, porcine reproductive and respiratory syndrome, transmissible gastroenteritis virus, vaccinia virus, feline calicivirus and, recently, coxsackievirus B3 (Skinner et al., 1980; Ziaie and Kefalides, 1989; Amsterdam et al., 1990; Gallicchio et al., 1993; Bschor, 1999; Everall et al., 2002; Harrison et al., 2007; Sui et al., 2010; Yamauchi and Helenius, 2013; Puertas et al., 2014; Ibáñez et al., 2018; Li et al., 2018; Zhao et al., 2020). Its ability to inhibit the growth of coronaviruses has also been demonstrated (Harrison et al., 2007) (Supplementary Table S1).

In the case of viral infections, Lithium exerts two types of actions; one is antiviral, and the other is its immunomodulatory effect; that is, the monovalent Li + cation exerts both neuroprotective and neurotrophic effects (Quiroz et al., 2010), which may result in immunoprotective and immunotrophic effects. The mechanism of action of lithium involves several signalling pathways such as inhibition of GSK3β (JA et al., 2004). GSK3 is an enzyme related to neuronal “pruning.” Its decrease results in increased neuronal retention. In addition, Li + ion acts competitively by binding directly to the Mg2+ binding sites of these enzymes (Hao et al., 2015). Thus, it is postulated that lithium may exert both beneficial actions to prevent poor prognosis in SARS-CoV-2 infection.

Considering its action on the immune system, lithium can mitigate the predominantly macrophagic and over-activated immune response responsible for the clinical deterioration and development of SARS. Secondly, lithium inhibits proinflammatory cytokines and will enhance the function of T lymphocytes to clear the virus from the body. In common parlance, it can change the “cytokine storm” to a “cytokine drizzle”. This led several groups to propose lithium as a possible treatment for SARS-CoV-2 (Gómez-Bernal, 2020; Murru et al., 2020; Spuch et al., 2020).

Based on these observations, we set out to conduct a clinical trial to evaluate the efficacy and safety of lithium treatment in patients infected with severe SARS-CoV-2.

## Materials and Methods

### Study Design

This clinical trial is a proof-of-concept study in which it is not necessary to make a formal sample size estimate. It is a prospective, longitudinal, interventional, and comparative study between a control group (*n* = 15), formed by patients with COVID-19 treated with standard treatment (dexamethasone 6 mg/24 h, 7–10 days) and an experimental group (*n* = 15), formed by patients with COVID-19 treated with standard treatment plus Lithium Carbonate (Plenur). Patients in the experimental group received 200 mg every 12 h of Lithium Carbonate, aiming for plasma levels between 0.6 and 1.2 mEq/L. In the following visits, depending on blood lithium levels, the dose could be modified, if necessary.

Patients with COVID-19 admitted to the Hospital Álvaro Cunqueiro (Vigo, Spain) were recruited. All of them were detected through the Microbiology Service by RT-PCR determinations for SARS-CoV-2. All patients who met all the inclusion criteria and none of the exclusion criteria were asked to sign the informed consent form. If obtained, 1:1 randomization was performed considering similar gender and age.

The primary outcome was assessed by measuring the number of days of hospital admission and the number of patients requiring admission to the ICU. Secondary outcomes were measured by assessing clinical and analytical parameters.

### Inclusion Criteria

Patients admitted to COVID-19 older than 18 years, who did not require an ICU at the time of inclusion in the study. Detection of SARS-CoV-2 RNA in nasopharyngeal samples was performed by quantitative real-time polymerase chain reaction (RT-PCR).

### Exclusion Criteria

Children under 18 years of age. Patients in whom lithium treatment was not appropriate due to their clinical condition (pregnancy and lactation; severe renal insufficiency, severe cardiovascular disease; severe dehydration or sodium depletion; hypersensitivity to lithium).

### Statistical Methods

Descriptive statistical analysis was performed for all variables. Continuous variables were summarized by N, mean, standard deviation (SD), median, maximum and minimum. On the other hand, categorical variables were described by N and the percentage of each category over the total valid values. In the case of missing values, their number per variable was described. Comparisons were performed using the χ2 test or the *t*-test as appropriate, or other statistical tests determined in the Statistical Analysis Plan. The significance level of all statistical tests was set at 0.05.

### Institutional Review Board Statement

The study was conducted in accordance with the guidelines of the Declaration of Helsinki and was approved by the Ethics Committee of the Galician Network of Research Ethics Committees (protocol code 2020/238, approval date 30/09/2020) and approval of the clinical trial by the AEMPS 06/06/2020 with number EudraCT 2020–002008–37. Written informed consent was obtained from all participants. All information collected was treated as strictly confidential, in accordance with national regulations.

### Plasma Isolation

Plasma samples were obtained using BD Vacutainer™ PET EDTA Tubes centrifugation, aliquoted, and cryopreserved at −80°C until further use.

### Cytokine Quantification in Plasma

Plasmas previously collected were used for the quantitative determination of cytokines (IL6, TNFα, IL2, IL10, IL12, IP10, IFNγ, sTIMP3, and IL1β by ELISA kits (Abyntek), and PD1-L1 by ELISA kit from Mybiosource according to the manufacturer’s instructions.

### Markers of Inflammation and Severity of Disease

Levels of CRP and absolute lymphocyte count were recorded from the electronic medical record.

### SARS-CoV-2 Viral Load Quantification

We performed nucleic acid extraction in a MicrolabStarlet IVD platform using the STARMag 96 × 4 universal Cartridge Kit (Seegene Inc., Seoul, South Korea). To detect SARS-CoV-2, we applied the Allplex™ SARS-CoV-2 Assay (Seegene Inc., Seoul, South Korea), a multiplex one-step rRT-PCR able to simultaneously detect four viral targets including the structural protein envelope (E) gene, the RNA-dependent RNA polymerase (RdRP) gene, the spike (S) gene, the nucleocapsid (N) gene, and an exogenous RNA-based internal control (IC). We targeted a conserved region in the structural protein envelope E-gene for pan-Sarbecovirus detection, RNA-dependent RNA polymerase (RdRP), and nucleocapsid (N) genes specific for SARS-CoV-2. For rRT-PCR, we employed the CFX96™ system (Bio-Rad Laboratories, Hercules, CA, United States). We analysed the results using Seegene Viewer-specific SARS-CoV-2 software (Seegene Inc., Seoul, South Korea). To establish a linear regression curve and obtain the concentration in copies/ml (inversely related to the cycle threshold value), the EDX SARS-CoV-2 Standard (Exact Diagnostics, TX, United States) containing 200,000 copies/mL of synthetic RNA transcripts from five gene targets (E, N, ORF1ab, RdRP and S Genes of SARS-CoV-2) was used.

### IgG SARS-CoV-2 Quantification

For serological evaluation of patients, we used two high throughput chemiluminescent immunoassay (CLIA) assays. First, LIAISONTM SARS-CoV-2 S1/S2 IgG (DiaSorin IgG; DiaSorin, Italy), that allows a semi-quantitative detection of IgG antibodies against to the S1 and S2 subunits of the Spike protein and second, ElecsysTM Anti-SARS-CoV-2 S (Roche T; Roche, United States), which allows the quantitative detection of total antibodies against the Spike protein.

## Results

A total of 30 patients (13 females and 17 males) were included in the study. Details of the characteristics of the patients in this study are presented in Table 1. The mean ± SD of age was 59.87 ± 18.28 (range 34–86) for the control group and 57.33 ± 16.29 (range 28–84) for the experimental group. The most common medical conditions were hypertension (30%), dyslipidaemia (20%), and obesity (16%). None of the patients in the lithium group had adverse effects associated with lithium carbonate medication (Supplementary Table S2).

**TABLE 1:** Characterization of the study group.

	Control Group	Lithium Group
Women, no. (%)	8 (53%)	5 (33%)
Age (years), mean ± SD	59.87 ± 18.28	57.33 ± 16.29
Age, 25% Percentile	43	47
Age, 75% Percentile	77	74
Age, range	34–86	28–84
SOFA scale, mean ± SD	0.87 ± 1.68	0.53 ± 1.12
pO2 (%), mean ± SD	94.87 ± 2.50	95.67 ± 2.58
Maximum blood pressure (mmHg), mean ± SD	128.07 ± 22.64	127.27 ± 18.70
Minimum blood pressure (mmHg), mean ± SD	76.33 ± 12.73	73.13 ± 11.65
Heart rate, mean ± SD	77 ± 12.78	79.07 ± 12.27
Comorbidities, number (%)		
Diabetes	0 (0%)	1 (3.33%)
High blood pressure	6 (20%)	3 (10%)
Obesity	3 (10%)	2 (6.67%)
Dyslipemia	3 (10%)	3 (10%)
Depression	2 (6.67%)	1 (3.33%)
Asthma	0 (0%)	1 (3.33%)
Cardiopathy	1 (3.33%)	1 (3.33%)
Myalgias	1 (3.33%)	0 (0%)
Osteoporosis	1 (3.33%)	0 (0%)

**FIGURE 1:**
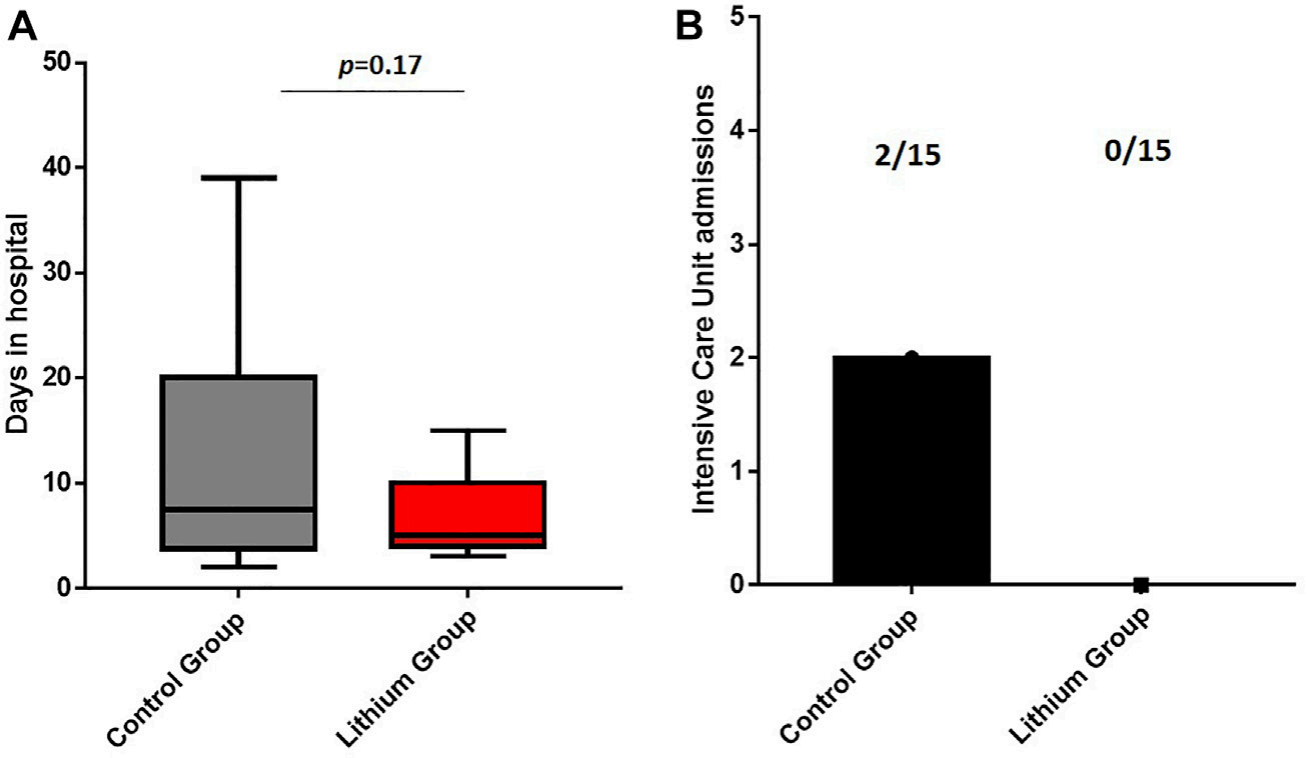
**(A)** Days of hospital admission. The lithium treatment group has a lower number of days of hospital admission. **(B)** Intensive Care Unit admissions. The control group had two intensive care unit admissions while the lithium group had no intensive care unit admissions.

Lithium treatment reduced hospital days; while the control group had a mean of 12.43 days (12.43 ± 12.09), the lithium group had a mean of 6.47 days (6.47 ± 3.60) (Figure 1). The difference is not statistically significant (*p* = 0.17) but shows a clear trend in the reduction of hospital admission days. None of the patients who received lithium required admission to the ICU, and none of them died. In contrast, in the control group, two patients were admitted to the ICU and one patient died (Figure 1).

**FIGURE 2:**
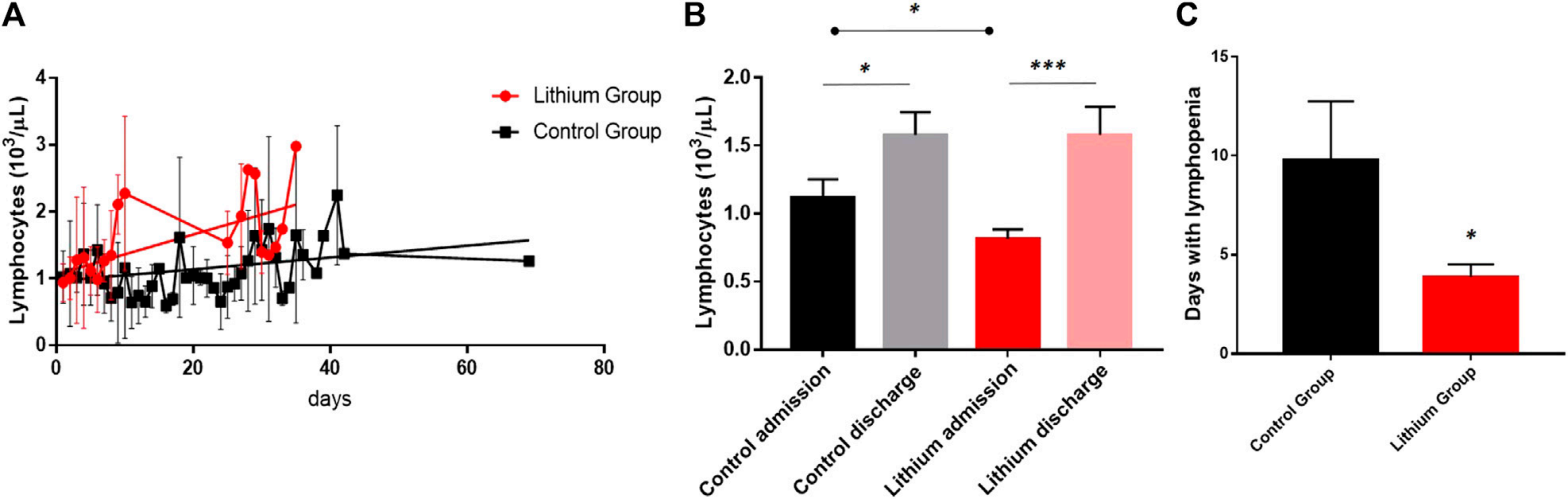
COVID-19 is characterized by very strong lymphopenia, and lithium is a modulator of the immune system. **(A)** In this graph we represent the mean values of lymphocytes in the two groups of the clinical trial, in red those treated with lithium and in black the control group. The lithium group recovered normal lymphocyte levels earlier than the control group. The lines show liner regression analysis, where a clear different pattern among groups is observed (*p* < 0.05) with different slopes for lithium group (red line, *R* ^2^ = 0.3379) and control group (black line, *R* ^2^ = 0.1711). **(B)** Although both groups recover from lymphopenia, the lithium group recovers significantly sooner by staying fewer days in lymphopenia. **(C)** The lithium treatment group remained significantly less days in lymphopenia than the control group (*p* < 0.05).

SARS-CoV-2 infection is characterized by marked lymphopenia, and since one of the properties of lithium is that it works as a good Immunomodulator, we investigated how it regulates blood lymphocyte levels. When we evaluated the levels of both groups during hospital admission, we clearly observed that lithium treatment significantly increased blood lymphocyte levels (Figure 2), although both groups at discharge ended up with normal blood lymphocyte levels, the lithium-treated patients recovered normal levels earlier. Patients in the control group remained 9.79 ± 11.14 days in lymphopenia, while patients on lithium treatment were only 3.87 ± 2.53 days in lymphopenia (Figure 2). With such a clear improvement in lymphocyte numbers, we decided to investigate whether lithium was able to modulate the amount of anti-SARS-CoV-2 antibodies generated after infection. We performed quantification by two different methods, the Liaison and the Roche methodology. With both methods we obtained similar results, and in both cases, there were no differences between the control group and the lithium group (Supplementary Figure S1).

**FIGURE 3:**
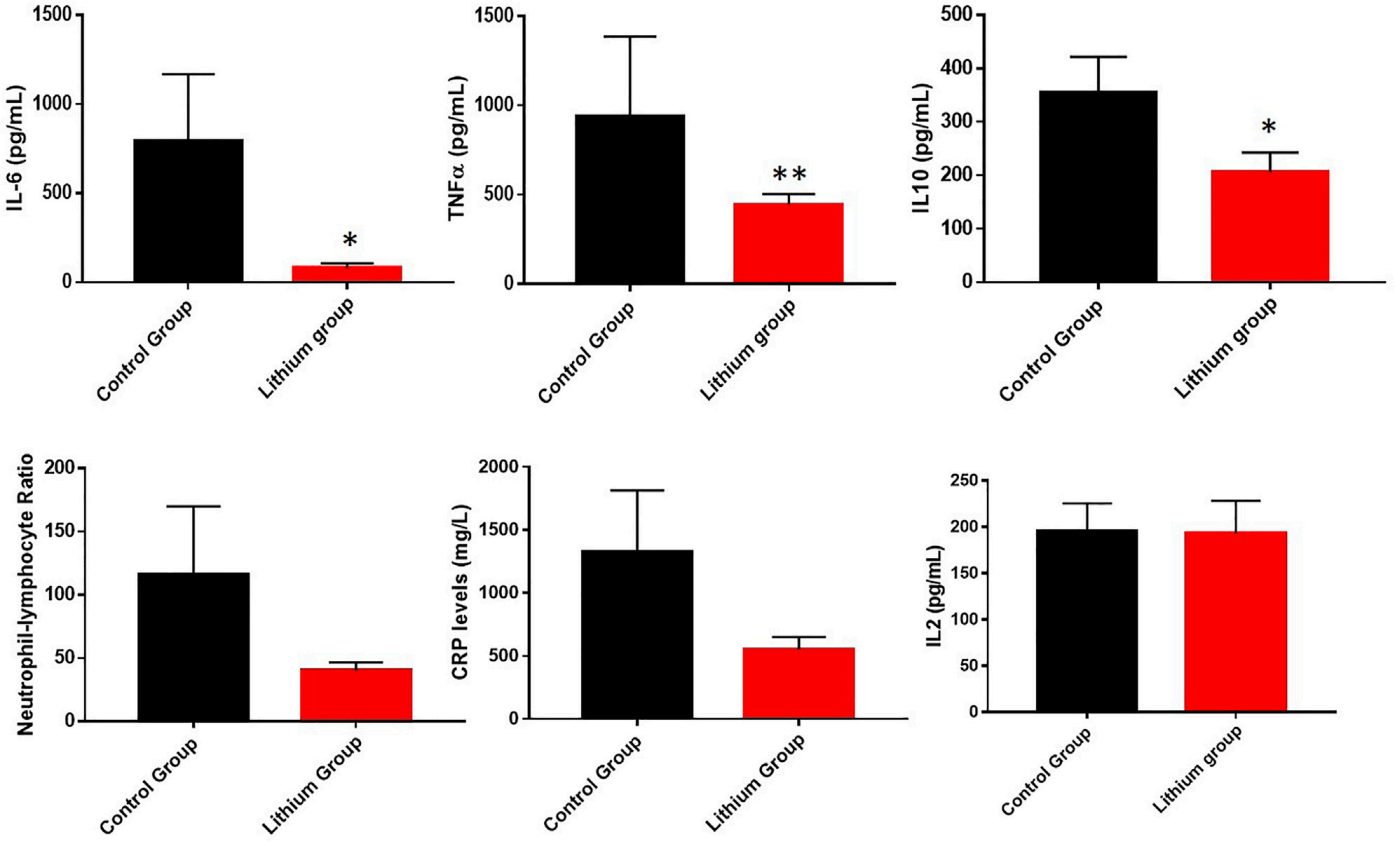
Representation of different parameters involved in general inflammation. We showed a reduction of general inflammation in the group of patients treated with lithium. The graphs represent the area under the curve of cytokine levels measured during the study. *p < 0.05 and **p < 0.01. Upper graphs represented the changes of IL-6, TNFα and IL-10 between control group and lithium group. The graphs below showed the area under the curve of the C-reactive protein graphs and neutrophil - lymphocyte ratio of all patients, control group and lithium group. We observed a tendency in the reduction of CRP levels and alower index of general inflammation without significance differences (*p* = 0.12 for NLR and *p* = 0.15 for CRP levels).

Next, we assessed the overall levels of inflammation. One of the characteristics of COVID-19 is the induction of an elevation of inflammatory parameters, strongly associated with disease severity (Tan C. et al., 2020; Tan et al., 2020 L.). In our study, we observed a favorable response of lithium treatment on inflammation, evidenced by a significant decrease in IL-6 levels (control group 792.1 ± 1128 vs. lithium group 77.83 ± 95.55, *p* < 0.05), TNFα (control group 952.6 ± 337.4 vs. lithium group 444 ± 59.1, *p* < 0.01) and IL-10 (control group 353.8 ± 68.21 vs. lithium group 206.2 ± 36.6, *p* < 0.05). In addition, clinical parameters on neutrophil-lymphocyte ratio (NLR) and C-reactive protein (CRP) levels, which are commonly used to detect general inflammation, were evaluated. In both cases, lithium treatment reduced the levels, not significantly but with a trend, with the NLR going from 116 ± 53.94 in the control group to 40.06 ± 6.544 in the lithium-treated group (*p* = 0.12). Something similar occurred with CRP levels, going from 1323 ± 491.4 in the control group to 549.7 ± 100.9 in the lithium-treated group (*p* = 0.15) (Figure 3).

**FIGURE 4:**
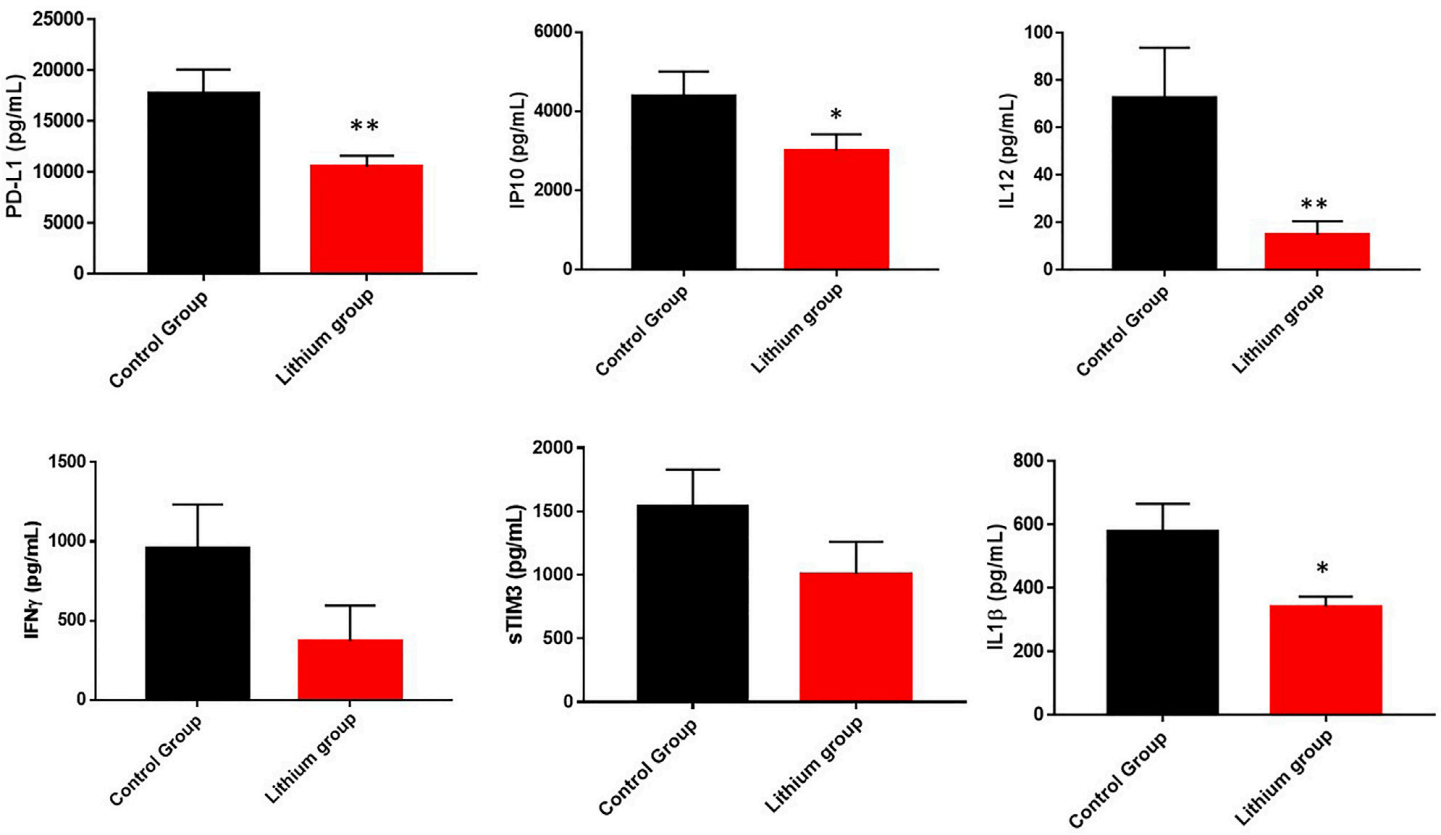
Representation of cytokine levels involved in acute inflammation (sTIM3, PD-L1, IP10, IL12, IFNγ and IL1β). These graphs showed the reduction of acute inflammation in the group of patients treated with lithium. The graphs represent the area under the curve of cytokine levels measured during the study. *p < 0.05 and **p < 0.01. In the case of IFNγ and sTIM3, there is a clear trend in the reduction of its levels in the group treated with lithium, although it is not significant (*p* = 0.10).

We also investigated in COVID-19 patients whose condition required ICU admission with severe acute inflammation whether lithium treatment was able to reduce cytokines involved in this level of inflammation. Indeed, lithium treatment was able to reduce the levels of most of the quantified cytokines (PD1-L1, IP10, IL-12, IL1b, IFNγ, and sTIMP3) (Figure 4). All decreased significantly, except IFNγ and sTIMP3, which showed a clear trend of decreasing levels, but the differences were not statistically significant. For IFNγ we went from 958 ± 277.6 in the control group to 374.2 ± 221.7 in the lithium-treated group (*p* = 0.10), for sTIMP3 we went from 1535 ± 296.3 in the control group to 1005 ± 255 in the lithium-treated group (*p* = 0.10).

**FIGURE 5:**
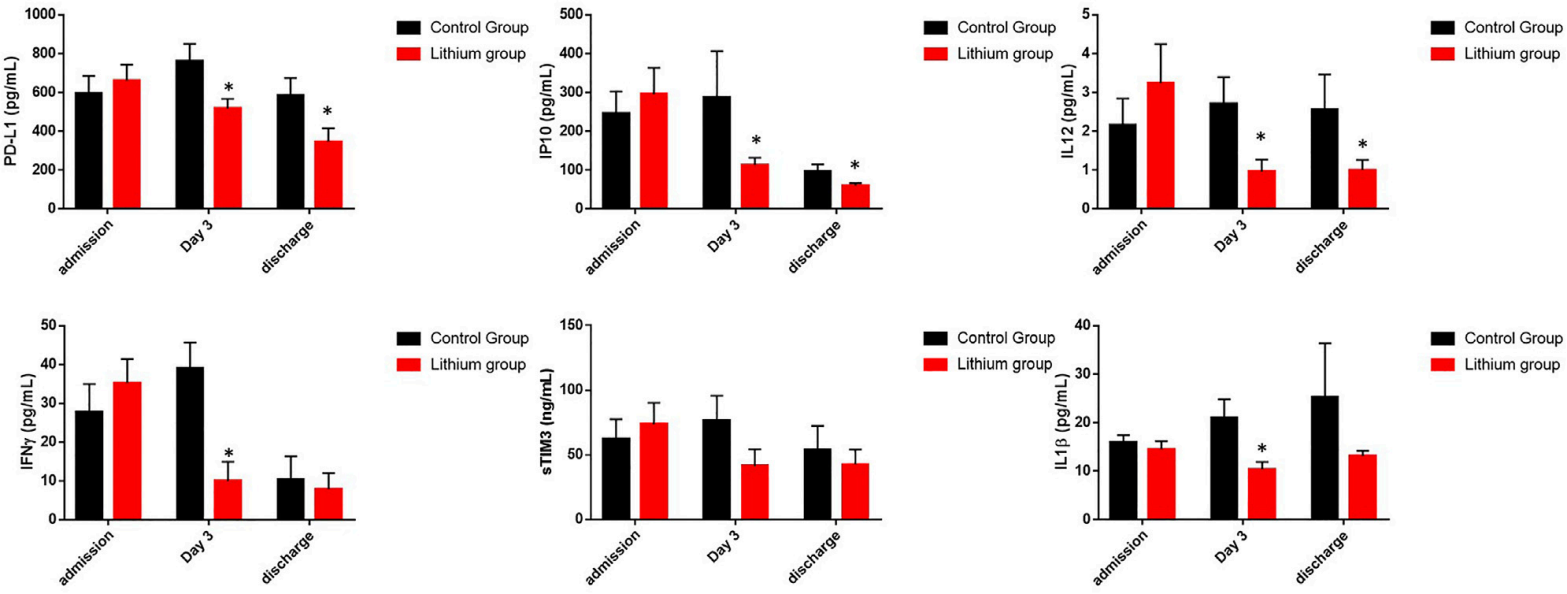
Representation of cytokine levels involved in acute inflammation measured during admission, after 3 days with treatment and during discharge. The graphs represent the area under the curve of cytokine levels measured during the study. *p < 0.05 and **p < 0.01. It can be seen how lithium treatment reduces the levels of acute inflammation after 3 days of treatment compared to the control group.

We chose to analyze the data on acute inflammatory cytokine levels temporally rather than by area under the curve during hospital admission. Temporally at admission, on day 3 (which is when the lithium treatment group generally had adequate pharmacological levels of lithium in the blood), and finally on the day of hospital discharge. On the day of hospital admission and acceptance to participate in the clinical trial, all patients had elevated inflammatory cytokines. However, on the third day, while the control group maintained elevated levels or worsened, the lithium treatment group improved significantly (Figure 5). This is important because it means that lithium may be reducing one of the main risk factors for poor patient outcomes.

**FIGURE 6:**
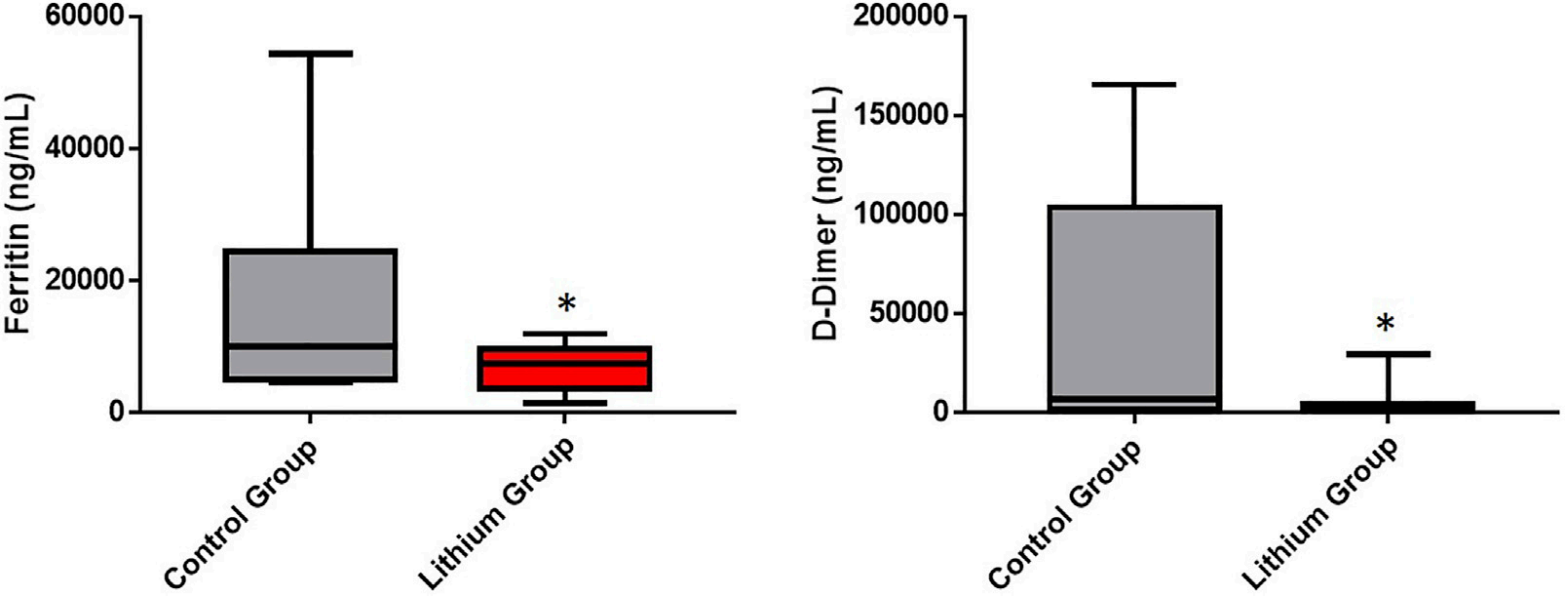
Reduction of ferritin and D-dimer levels in patients treated with lithium. The area under the curve of the ferritin and D-dimer levels of patients, control group and lithium group is quantified. Ferritin is an index that indicates the accumulation of iron and is associated with the inflammatory activity of macrophages. Lithium reduces significantly the levels of ferritin (*p* < 0.05). COVID-19 is usually complicated by coagulopathy. D-dimer is a maker of thrombin generation and fibrinolysis and this constitutes a relevant prognostic index of mortality from the infection. The D-dimer value is associated with the severity of patients with COVID-19. Lithium reduces significantly D-dimer levels and the risk of coagulopathy and mortality (*p* < 0.05).

In addition, two clinical aspects that help predict whether the SARS-CoV-2 infected patient will have severe problems during admission are ferritin and D-dimer levels. We know that increased serum ferritin and D-dimer during admission predicts mortality of COVID-19 patients in the intensive care unit. In our study we observed how lithium treatment significantly reduced ferritin and D-dimer levels (Figure 6). During hospital admission both groups started from similar levels of ferritin (control group: 561.1 ± 615.20 ng/ml and lithium group: 605.5 ± 546.24 ng/ml, *p* < 0.05) and D-dimer (control group: 3,260.22 ± 7,346.82 and lithium group: 460.55 ± 185.46 ng/ml, *p* < 0.05). However, while the control group maintained elevated levels of both proteins, the lithium-treated group strongly reduced their blood levels (Figure 6).

Since lithium has shown antiviral properties against various types of viruses, we set out to investigate whether patients on lithium treatment reduced viral load levels with respect to the control. We quantified the viral load by means of nasopharyngeal samples on the day of hospital admission and the day of hospital discharge. In this case, we found no differences between the two groups (Supplementary Figure S2).

## Discussion

COVID-19 is an infection caused by the SARS-CoV-2 virus to which the immune system reacts in two phases. The first is the activation of the innate immune response, during which innate immune cells secrete interferons and cytokines to elicit the recruitment of macrophages, monocytes, and neutrophils that act as the first line of defence (Andrews et al., 2010). While this phase is developing, the second line of defence is activated, which is the adaptive immune response, activating T lymphocytes that stimulate B lymphocytes to produce specific antibodies against the virus.

One of the pathophysiological problems of COVID-19 is that it produces a maladaptive or hyper-reactive immune response, in which innate cell lineages (macrophages and neutrophils) are greatly increased, and lymphocytes greatly reduced (lymphopenia). This is associated with increased disease severity and worse prognosis, ultimately leading to intensive interventions with a high risk of intubation and mortality (Lucas et al., 2020; Qin et al., 2020).

Lithium is a drug that is part of a class of drugs called antimanic agents. It is used in the form of lithium carbonate as a mood stabilizer in people with bipolar disorder, and in Spain, it is marketed under the brand name Plenur. Lithium is used to treat and prevent episodes of mania (a frenzied and abnormally excited mood with grandiose and/or irritable behavior) in people with bipolar disorder (a disease that causes episodes of depression, suicide, mania, and other abnormal moods), and is also sometimes used in the treatment of resistant depression, schizophrenia, impulse control disorders or agitation in people with intellectual disability. It is the most effective drug in reducing suicide attempts and completed suicides (Won and Kim, 2017).

The mechanism of action of lithium is increasingly well known. It is now known that lithium exerts its action on the nervous system, specifically on brain cell retention, inhibiting glycogen synthase kinase 3β (GSK3 β) and improving conduction. As a result, neurotrophic factors, neurotransmitters, oxidative metabolism, apoptosis, and second messenger systems are modulated (Castillo-Quan et al., 2016). In simple terms, this translates into a reduction in brain aging over time. Because of these broad benefits in the CNS, lithium has also been described to have poorly understood functions in other tissues. For example, the positive effects of lithium use on various components of human blood; including, but not limited to, hematopoietic stem cells, neutrophil count, granulocyte count, thrombocyte count, dendritic cell count, and monocyte count, and on the functioning of the immune system itself through the regulation of B and T lymphocytes (Gallicchio and Chen, 1981; Barr and Galbraith, 1983; Duvall and Gallicchio, 2017). Another study shows that lithium can inhibit NF κβ nuclear translocation in macrophages and modulate the inflammatory profile after Rift Valley fever virus infection (Makola et al., 2021). Evidence was found that lithium was able to regulate both B and T lymphocytes. Interestingly, in a study on patients with thyroid hormonal imbalances, lithium was shown to be able to increase the activity of B lymphocytes and reduce the ratio of circulating suppressor T cells to cytotoxic T cells (Kibirige et al., 2013). In another recent work, lithium showed an antiapoptotic effect on T lymphocytes in patients with bipolar disorder (Pietruczuk et al., 2018).

Early in the pandemic, we began to observe that the immune system response was one of the causes of severe symptoms in COVID-19. Based on these known actions of lithium on the immune system, we conducted an observational study of six patients with bipolar disorder already taking lithium admitted to the ICU. We observed a strong improvement, especially in their inflammatory parameters (Spuch et al., 2020), a less aggressive reactivity and a more adapted response. Other comments in different publications raised the same hypothesis on the use of lithium to reduce severe symptoms caused by COVID-19 (Gómez-Bernal, 2020; Rudd, 2020; Khosravi, 2021). Based on these preliminary data, we decided to perform a randomized clinical trial with 30 patients with severe COVID-19 (EudraCT number 2020–002008–37) with two arms, a control arm with the usual treatment, in this case corticosteroid treatment, and the treatment arm with lithium added to the usual treatment. After the clinical trial, we observed that lithium treatment reduced the number of days of hospitalization, showing a clear trend. It reduced ICU (intensive care unit) admissions and death. In the control group, two patients were admitted to the ICU and one of them died, while in the lithium treatment arm, none were admitted to the ICU.

Increasing evidence points to an association between immune response and disease progression in COVID-19, in which unknown abnormalities in immune cells appear to be responsible for the uncontrolled elevation of inflammatory markers and subsequent cytokine storm, leading to disease severity and fatal outcome (Lucas et al., 2020). Therefore, finding a treatment that modulates the immune system would be key to reducing severe disease symptoms and thereby reducing ICU admission and risk of death. A treatment that is ubiquitous, inexpensive and effective would be even more valuable. COVID-19 causes a sharp drop in lymphocyte levels, which persists for many days during the disease. Several studies have found a correlation between lymphopenia and disease severity, a condition defined by abnormally low lymphocyte counts (Tan L. et al., 2020; Fathi and Rezaei, 2020; Tavakolpour et al., 2020). In our clinical trial, lithium increases lymphocyte levels much more rapidly, and lithium patients remain lymphogenic for fewer days than controls. A recent study shows that SARS-CoV-2 mainly affects lymphocytes and not innate cells (Ekşioğlu-Demiralp et al., 2021), resulting in a dysregulation of the system between the adaptive and innate immune systems. Our data show that lithium is a good treatment to rapidly increase lymphocyte levels and prevent the severity of infection.

Inflammation is a key feature of SARS-CoV-2 infection. When patients have an excessive inflammatory response, this can lead to unfavorable processes and even death. Although many infected patients are asymptomatic or have only mild or moderate symptoms, a small minority of patients develop the severe or life-threatening disease (Wong, 2020). This is because the very effects of the exacerbated immune response are more destructive than those of the infection itself, causing the so-called cytokine storm, which results in massive and irreversible damage to many organs such as the lung (Costela-Ruiz et al., 2020). When we analyzed the general parameters of inflammation in our patients, we observed elevated levels of TNF-α, IL-6, IL-10, neutrophil-lymphocyte ratio and C-reactive protein (CRP) levels. When we treated them with lithium, we observed that all general inflammation parameters were reduced. These elevated parameters, as well as lymphopenia, are a risk factor for COVID-19 morbidity and mortality, implying that lithium treatment would reduce these parameters leading to a decreased risk of disease severity.

In addition to general inflammation parameters related to cytokine storm, we decided to investigate how lithium affects different molecules related to acute inflammation and the regulation of the immune system itself, such as sTIM3 (soluble T-cell immunoglobulin mucin domain), PD-L1, IP10, IL12, IFNγ and IL1 β. Immune checkpoint molecules, such as PD-L1, sTIM3-3 and IFNγ, play an important role in regulating the host immune response and, for unknown reasons, are down-regulated in COVID-19 (Sabbatino et al., 2021; Ueland et al., 2021). IP-10, also known as CXCL10, is a proinflammatory cytokine secreted by immune cells. Together with IL12 and IL1 β it is associated with defence against various infections being highly elevated in patients with COVID-19 (Hashimoto et al., 2021; Kesmez Can et al., 2021). When we treated patients with lithium, we observed that PD-L1, IP10, IL12 and IL1 β levels were significantly reduced. In the case of IFNγ and sTIM3 values, although they are reduced with lithium treatment, it is not statistically significant. These data are evident when we calculate the levels of these proteins on the day of hospital admission, on the third day, which is when they already have lithium treatment at pharmacological doses, and on the day of hospital discharge. It was observed that on the third day all the levels of these cytokines and immune regulators were reduced, remaining constant until hospital discharge. Thus, it can be deduced that, although we do not know the mechanisms of lithium regulation on this part of the immune system, it does indicate that lithium is modulating the checkpoint mechanisms of the immune system, as well as regulating the mechanism of T lymphocyte suppression.

In addition, ferritin and D-dimer levels (two very important clinical parameters implicated in the severity of COVID-19 and in the cause of severe problems after discharge - “long COVID"-) improve in patients with lithium treatment. Ferritin is a protein related to the acute phase of immune system activation and a parameter of macrophage activity. As a hallmark of hyperferritinemic syndromes, elevated circulating ferritin is found in four critical diseases: macrophage activation syndrome (MAS), adult-onset Still’s disease (AOSD), catastrophic antiphospholipid syndrome (CAPS), and septic shock. Excessive elevation of ferritin was associated early in the pandemic with severity and high mortality risk in COVID-19 patients (Kaushal et al., 2021). D-dimer level is one of the common measures used in patients to detect thrombosis in patients with coagulopathy problems. D-dimer levels are known to be elevated in the early stages of infection and high levels are associated with poor prognosis (Rostami and Mansouritorghabeh, 2020; Terpos et al., 2020).

On the other hand, one of the most promising actions of lithium is the evidence of its action on multiple viral infections. There is evidence that lithium can inhibit the replication of several viruses, some similar and belonging to the coronavirus family (Skinner et al., 1980; Hao et al., 2015; Zhou et al., 2015). One of the objectives of this clinical trial was to determine whether lithium treatment was able to reduce viral load within a few days. However, due to methodological problems arising from clinical care due to the pandemic, it was only possible to determine the viral load on the day of admission and the day of hospital discharge. At these times there were no differences between the control and lithium treatment groups. The incubation period for COVID-19 is thought to be between 2 and 14 days after exposure, with most cases presenting symptoms approximately 4–5 days after exposure (Lauer et al., 2020), which is one of the limitations of the study, since the changes that might occur in viral load should occur during these first 2 weeks of treatment.

Another factor we wanted to determine was whether lithium treatment was able to improve the response to anti-SARS-CoV-2 antibody generation after hospital discharge (1 month after the start of the clinical trial). We performed it with two different methodologies since they were new techniques that were being introduced in hospital laboratories. We detected no differences between the two groups, indicating that survivors of infection develop optimal antibody levels.

Finally, one data that reaffirms the improvement of lithium-treated patients is that when we examined patients 1 month after hospital discharge and long-term neurological effects were recorded, 40% of lithium-treated patients were observed to have symptoms, while 73% of the control group had some neurological symptoms (Table 2). These data deserve to be investigated with a large number of patients since the reduction of neuropsychiatric symptoms of “long COVID-19” would mean a substantial improvement in the health status of the recovered patients.

**TABLE 2:** Neurological long-term effects 1 month after discharge.

Lithium Group	Long Term Effects (1 month after Discharge)
Patient 1	Brain fog
Patient 3	None
Patient 5	None
Patient 7	None
Patient 9	Headache
Patient 11	Myalgia
Patient 13	None
Patient 15	Brain fog, asthenia and dyspnea
Patient 17	None
Patient 19	None
Patient 21	None
Patient 23	Brain fog, dyspnea and anosmia
Patient 23	Dyspnea and migraine
Patient 25	Asthenia and dyspnea
Patient 27	None
Patient 29	None
**Control Group**	**Long Term Effects (1 month after Discharge)**
Patient 2	admitted to the ICU
Patient 4	Hearing loss and difficulty in ambulation
Patient 6	Headache and myalgia
Patient 8	Dyspnea and reduced mobility
Patient 10	None
Patient 12	Asthenia, myalgia and dyspnea
Patient 14	Asthenia, myalgia and dyspnea
Patient 16	None
Patient 18	None
Patient 20	Asthenia and dyspnea
Patient 22	Brain fog, asthenia and dyspnea
Patient 24	Dyspnea and migraine
Patient 26	None
Patient 28	Brain foggy and headache
Patient 30	Admitted to the ICU and death

## Conclusion

This randomized clinical trial demonstrates that lithium carbonate treatment is safe and effective in curbing the severity caused by SARS-CoV-2 infection. SARS-CoV-2 infection induces exaggerated inflammation driven by components of innate immunity. We demonstrated that lithium was able to reduce the number of days of hospital and ICU admission as well as the risk of death. Lithium, through its immunomodulatory action, reduces inflammatory cytokine levels by preventing cytokine storms, thus reducing the severity of the infection and the risk of death. In fact, lithium can also be studied for earlier use, such as at the time of diagnosis, in order to avoid hospitalization altogether, as well as in the treatment of “Long Covid” syndromes. From the “Big Bang” to its presence in all living things, to its use in medicine, and in the storage of electricity, the third element of the Periodic Table may be paramount in life, which cannot be spelled without Li.

This clinical trial demonstrates that lithium deserves to be investigated in-depth and with a larger number of patients to treat COVID-19 in severe patients.

## Data Availability

The raw data supporting the conclusions of this article will be made available by the authors, without undue reservation.
