# Supplementary material for: Efficacy and Safety of Lithium Treatment in SARS-CoV-2 Infected Patients
Source: Front Pharmacol. 2022 Apr 14;13:850583. doi: 10.3389/fphar.2022.850583 (PMC9046673; doi:10.3389/fphar.2022.850583)
Supplement: Supplementary file 1 [file Table1.docx]

| **Reference** | **Family** | **Genus** | **Virus** | **Infection** | **Genome** | **Experiment** |
| --- | --- | --- | --- | --- | --- | --- |
| **Zhao et al., 2020** | Picornaviridae | Enterovirus | B3 coxsackievirus (CVB3) | Viral myocarditis | RNA (7.4 kb) | Mice  HeLa cells  293T cells |
| **Zhai et al., 2019** | Coronaviridae | Deltacoronavirus | Porcine deltacoronavirus (PDCoV) | Porcine infection | RNA (25 kb) | LLC-PK1 cells |
| **Li et al., 2018** | Coronaviridae | Aphacoronavirus | Porcine epidemic diarrhea virus (PEDV) | Porcine epidemic diarrhea (PED) | RNA (28 kb) | Vero cells |
| **Chen et al., 2016** | Reoviridae | Orthoreovirus | Mammalian orthoreovirus (MRV) | Reovirus infection | RNA (23.5 kb) | Vero cells |
| **Zhou et al., 2015** | Parvoviridae | Protoparvovirus | Canine parvovirus (CPV) | Canine parvovirus type 2 (CPV-2) infection | DNA (5.2 kb) | Feline kidney cells (F81) |
| **Chen et al., 2015** | Parvoviridae | Protoparvovirus | Porcine parvovirus (PPV) | PPV infection | DNA (5 kb) | Swine testis cells |
| **Cui et al., 2015** | Arteriviridae | Betaarterivirus | Porcine reproductive and respiratory syndrome virus (PRRSV) | PRRS infection | RNA (14.9 – 15.5 kb) | MARC-145 cells |
| **Hao et al., 2015** | Arteriviridae | Betaarterivirus | Porcine reproductive and respiratory syndrome virus (PRRSV) | PRRS infection | RNA (14.9 – 15.5 kb) | MARC-145 cells |
| **Puertas et al., 2014** | Reoviridae | Lentivirus | Human immunodeficiency virus (HIV) | Human acquire immunodeficiency syndrome (AIDS) | RNA (9 kb) | Humans |
| **Ren et al., 2011** | Coronaviridae | Alphacoronavirus | Transmissible gastroenteritis virus (TGEV) | Enteric infection | RNA (28.5 kb) | Swine testis cells  Porcine kidney cell (PK-15) |
| **Sui et al., 2010** | Herpesviridae | Varicellovirus | Pseudorabies virus (PrV) | PrV infection | DNA (145 kb) | Vero cells |
| **Li et al., 2009** | Coronaviridae | Gammacoronavirus | Avian infectious bronchitis virus (IBV) | IBV infection | RNA (27.6 kb) | Vero cells |
| **Ling et al., 2009** | Reoviridae | Lentivirus | Human immunodeficiency virus (HIV) | Human acquire immunodeficiency syndrome (AIDS) | RNA (9 kb) | Mice |
| **Harrison et al., 2007** | Coronaviridae | Gammacoronavirus | Avian infectious bronchitis virus (IBV) | IBV infection | RNA (27.6 kb) | Cells |
| **Dou et al., 2005** | Reoviridae | Lentivirus | Human immunodeficiency virus (HIV) | Human acquire immunodeficiency syndrome (AIDS) | RNA (9 kb) | Human fetal neurons |
| **Everall et al., 2002** | Reoviridae | Lentivirus | Human immunodeficiency virus (HIV) | Human acquire immunodeficiency syndrome (AIDS) | RNA (9 kb) | Mice  SH-SY5Y neuronal cells |
| **Bschor, 1999** | Herpesviridae | Simplexvirus | Herpes simplex virus (HSV) | Herpes labialis | DNA (152 kb) | Humans |
| **Gallicchio et al., 1993** | Reoviridae | Lentivirus | Human immunodeficiency virus (HIV) | Human acquire immunodeficiency syndrome (AIDS) | RNA (9 kb) | Mice |
| **Amsterdam et al., 1990** | Herpesviridae | Simplexvirus | Herpes simplex virus (HSV) | HSV infection | DNA (152 kb) | Humans |
| **Ziaie et al., 1989** | Herpesviridae | Simplexvirus | Herpes simplex virus type 1 (HSV-1) | HSV-1 infection | DNA (152 kb) | Human umbilical vein endothelial cells |

Supplementary Table 1: Antiviral effects of lithium salts over the years.

Legend: Review of literature based on studies published until 02/04/2020 with the search terms: “lithium” and “antiviral”; “lithium” and “antiretroviral”; or “lithium” and “viral” in the PubMed database
