# Supplementary figures and images for: Efficacy and Safety of Lithium Treatment in SARS-CoV-2 Infected Patients
Source: Front Pharmacol. 2022 Apr 14;13:850583. doi: 10.3389/fphar.2022.850583 (PMC9046673; doi:10.3389/fphar.2022.850583)

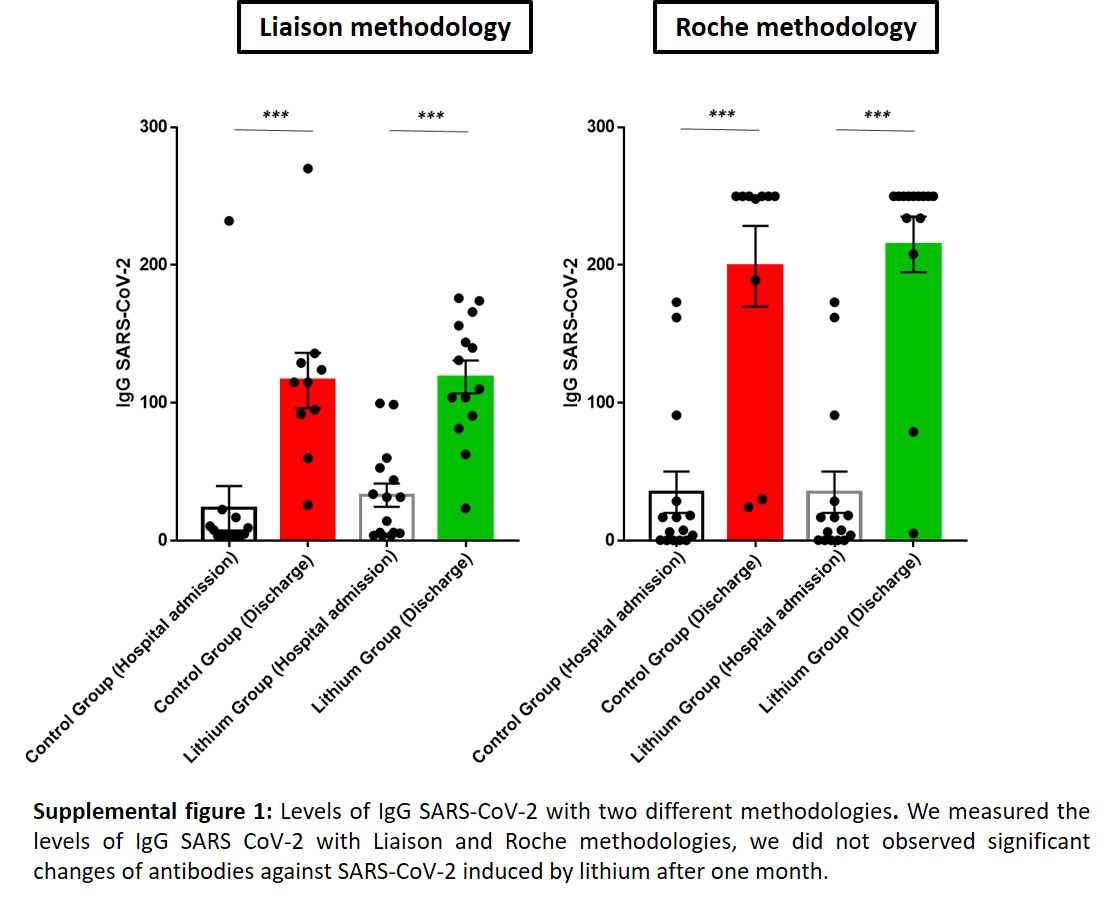

Supplement: Supplementary file 2 [file Image1.JPEG]

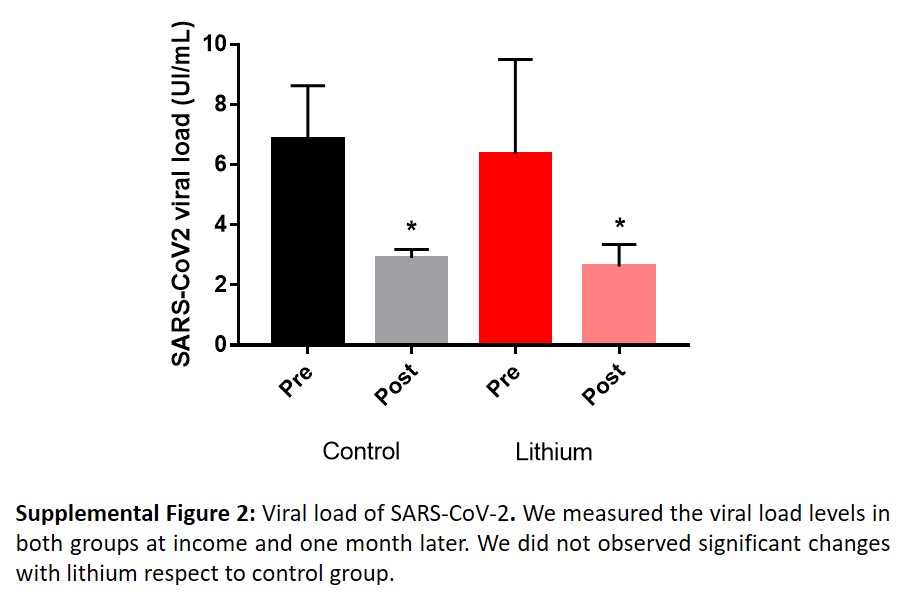

Supplement: Supplementary file 3 [file Image2.JPEG]
