# Supplementary material for: Efficacy and Safety of Lithium Treatment in SARS-CoV-2 Infected Patients
Source: Front Pharmacol. 2022 Apr 14;13:850583. doi: 10.3389/fphar.2022.850583 (PMC9046673; doi:10.3389/fphar.2022.850583)
Supplement: Supplementary file 4 [file Table2.docx]

| **Lithium group** | **Medication** |
| --- | --- |
| Patient 1 | Methylprednisolone 120mg/24h, lithium 200mg/12h |
| Patient 3 | Methylprednisolone 20mg/8h, Amitriptyline 10mg/24h, lithium 200mg/12h |
| Patient 5 | Methylprednisolone 250mg/24h, lithium 200mg/12h |
| Patient 7 | Methylprednisolone 250mg/24h, azithromycin 500mg/24h, lithium 200mg/12h |
| Patient 9 | Tramadol 50mg/24h, dexamethasone 4mg/12h, lithium 200mg/12h |
| Patient 11 | lithium 200mg/12h |
| Patient 13 | lithium 200mg/12h |
| Patient 15 | Methylprednisolone 250mg/24h, azithromycin 500mg/24h, dexamethasone 4mg/12h, lithium 200mg/12h |
| Patient 17 | Methylprednisolone 250mg/24h, lithium 200mg/12h |
| Patient 19 | Amoxicillin/clavulanate 200mg/8h, lithium 200mg/12h |
| Patient 21 | Methylprednisolone 250mg/24h, lithium 200mg/12h |
| Patient 23 | Methylprednisolone 250mg/24h, lithium 200mg/12h |
| Patient 25 | Lithium 200mg/12h |
| Patient 27 | Methylprednisolone 250mg/24h, lithium 200mg/12h |
| Patient 29 | Lithium 200mg/12h |
| **Control group** | **Medication** |
| Patient 2 | Methylprednisolone 120mg/24h, azithromycin 500mg/24h |
| Patient 4 | Methylprednisolone 120mg/24h |
| Patient 6 | Alprazolam 0.5mg/24h, pregabalin 75mg/24h, tramadol 50mg/24h |
| Patient 8 | Methylprednisolone 120mg/24h, Quetiapine 25mh/24h, haloperidol 5mg/24h, duloxetine 60mh/24h, dexamethasone 4mg/8h |
| Patient 10 | Duloxetine 60mg/24h, amoxicillin/clavulanate 200mg/8h |
| Patient 12 | Methylprednisolone 120mg/24h, amoxicillin/clavulanate 200mg/8h |
| Patient 14 | Methylprednisolone 120mg/24h |
| Patient 16 |  |
| Patient 18 |  |
| Patient 20 |  |
| Patient 22 | Methylprednisolone 250mg/24h |
| Patient 24 | Methylprednisolone 250mg/24h |
| Patient 26 |  |
| Patient 28 | Methylprednisolone 250mg/24h |
| Patient 30 | Methylprednisolone 250mg/24h, Vancomycin, Linezolid, ceftazidime with avibactam |

**Supplementary Table S2**: Medication of clinical trial patients
